# ASO Author Reflections**: Accurate Evaluation of Malignant Potential in Pancreatic Ductal Adenocarcinoma Using DUPAN-2 Level**

**DOI:** 10.1245/s10434-024-15272-2

**Published:** 2024-05-13

**Authors:** Tatsuaki Sumiyoshi, Kenichiro Uemura

**Affiliations:** Department of Surgeryof Biomedical and Health Science, Hiroshima University, Hiroshima, Japan

## Past

Patients with pancreatic ductal adenocarcinoma (PDAC) with a Lewis antigen-negative phenotype secrete very little or no carbohydrate antigen (CA) 19-9. Therefore, PDAC patients with normal CA 19-9 levels can have early-stage cancer or advanced cancer without elevation of CA19-9 level; thus, estimating their malignant potential is difficult.

## Present

The combined use of CA19-9 and Duke pancreatic monoclonal antigen type 2 (DUPAN-2) levels may be useful in estimating the real malignant potential in all patients with PDAC because DUPAN-2 levels do not depend on the Lewis antigen phenotype.^1,2^

## Future

The combined use of CA19-9 and DUPAN-2 may define a novel criterion of borderline resectability.
